# Sialic-Acid-Related Enzymes of B Cells and Monocytes as Novel Markers to Discriminate Improvement Categories and to Fulfill Two Remission Definitions in Rheumatoid Arthritis

**DOI:** 10.3390/ijms241612998

**Published:** 2023-08-20

**Authors:** Lieh-Bang Liou, Ping-Han Tsai, Yao-Fan Fang, Yen-Fu Chen, Chih-Chieh Chen, Jenn-Haung Lai

**Affiliations:** 1 Division of Rheumatology, Allergy and Immunology, New Taipei Municipal Tucheng Hospital, New Taipei City 236, Taiwan; s001033@gmail.com (P.-H.T.); pisces4018@cgmh.org.tw (C.-C.C.); 2 Division of Rheumatology, Allergy and Immunology, Chang Gung Memorial Hospital at Linkou, Taoyuan 333, Taiwan; 8802012@cgmh.org.tw (Y.-F.F.); patrichen0693@gmail.com (Y.-F.C.); laiandho@gmail.com (J.-H.L.); 3 School of Medicine, Chang Gung University College of Medicine, Taoyuan 333, Taiwan

**Keywords:** Sialyltransferases, neuraminidases, B cells, monocytes, improvement criteria for RA, remission definitions for RA

## Abstract

The enzymes α-2,6-sialyltransferase 1 (ST6Gal1), neuraminidase 1 (Neu1), α-2,3-sialyltransferase 1 (ST3Gal1), and neuraminidase 3 (Neu3) are known to affect immune cell function. However, it is not known whether the levels of these enzymes relate to remission definitions or differentiate American College of Rheumatology (ACR), European League Against Rheumatism (EULAR), and Simplified Disease Activity Index (SDAI) responses in patients with rheumatoid arthritis (RA). We measured the ST6Gal1, Neu1, ST3Gal1, and Neu3 levels of B cells and monocytes in RA patients and correlated the cells’ enzyme levels/ratios with the improvement in the ACR, EULAR and SDAI responses and with the two remission definitions. The difference in the B-cell Neu1 levels differed between the ACR 70% improvement and non-improvement groups (*p* = 0.043), between the EULAR good major response (improvement) and non-good response groups (*p* = 0.014), and also between the SDAI 50% or 70% improvement and non-improvement groups (*p* = 0.001 and 0.018, respectively). The same held true when the RA patients were classified by positive rheumatoid factor or the use of biologics. The B-cell Neu1 levels significantly indicated 2005 modified American Rheumatism Association and 2011 ACR/EULAR remission definitions (area under the curve (AUC) = 0.674 with *p* = 0.001, and AUC = 0.682 with *p* < 0.001, respectively) in contrast to the CRP and ESR (all AUCs < 0.420). We suggest that B-cell Neu1 is superior for discriminating ACR, EULAR, and SDAI improvement and is good for predicting two kinds of remission definitions.

## 1. Introduction

The Disease Activity Score 28 (DAS28-erythrocyte sedimentation rate (ESR)) has been used regularly in clinical practice to monitor rheumatoid arthritis (RA) disease activity [1]. Although it is not perfect [2], the DAS28 is used worldwide, and the DAS28-ESR cutoff of 2.6 defines a low disease activity state, previously—when only conventional synthetic disease-modifying anti-rheumatic drugs (DMARDs) were available on the market—this state was known as “remission”. Furthermore, when compared with the DAS28-ESR, the DAS28-C-reactive protein (CRP) notably underestimated RA disease activity [3]. In particular, patients with active RA may have normal levels of blood inflammatory markers (ESR/CRP) and vice versa [4,5]. Interestingly, plasma monocyte chemotactic protein-1 (MCP-1) is correlated with two elements of the DAS28-ESR (the swollen joint count (SJC) and tender joint count (TJC)); on the contrary, the ESR and CRP are correlated with only one element, the SJC [6]. The ESR and CRP levels represent different underlying pathophysiologies, and the DAS28-CRP threshold values differ from those of DAS28-ESR [7]. DAS28-ESR and DAS28-CRP are used only for assessing RA disease activity as described [6,7,8,9]. Similarly, the α-2,6-sialic acid (SIA), sialyltransferase (ST), and neuraminidase (Neu) levels of immune cells might represent underlying pathophysiologies different from those of the ESR and CRP (see below). Therefore, other laboratory biomarkers are potentially useful in predicting remission and improvement categories in daily clinical practice.

Many molecules have been implicated in inducing pathological changes and indicating clinical disease activity in RA. However, the correlation between the SIA and SIA-related enzyme levels of monocytes and RA disease activity is not fully understood. A group of reports imply that the phagocytes of antigen-presenting cells with high cell-surface SIA levels are immunologically immature, displaying less phagocytosis or less IgG binding [10,11,12]. Cell SIA is known to be regulated by both ST and Neu (see below).

The association between levels of α-2,6-ST I (ST6Gal1) and RA pathogenesis has been examined by various researchers. For example, low α-2,6-sialylated immunoglobulin G (IgG) Fc fragments of anti-collagen antibodies and anti-citrullinated protein antibodies (ACPA) were observed in collagen-induced arthritis (CIA) in mice and in RA patients, respectively [13]. In particular, *st6gal1* gene-depleted mice produced low α-2,6-sialylated IgG anti-collagen antibodies that resulted in exacerbated joint inflammation and high arthritis scores. In contrast, highly sialylated ACPA (through the transfected *st6gal1* gene) was protective in contrast to control ACPA in collagen antibody-induced arthritis [13]. Similarly, low-sialylated synovial fibroblasts were detected in the joints of CIA mice and of active RA patients [14]. In contrast, highly sialylated synovial fibroblasts became dominant in RA patients in remission. That is, transformation of low sialylated synovial fibroblasts into high sialylated ones is congruent with transformation of active RA into RA in remission. Moreover, ST6Gal1 depletion in synovial fibroblasts by small interfering RNA increased IL-6 and MCP-1 secretion, suggestive of pro-inflammatory status. Tumor necrosis factor inhibited expression of *st6gal1* and α-2,6-sialylation in fibroblasts from mice [14]. Therefore, not only is the content of α-2,6-SIA in autoantibodies important for pathogenesis of human RA and CIA in mice, but ST6Gal1 and Neu1 capable of producing and removing α-2,6-SIA are also important for collagen-induced arthritis in mouse and human fibroblasts.

Sialyltransferases transfer SIA to a nascent glycoprotein or glycolipid [15,16]. ST6Gal1 transfers the α-2,6-SIA residue to a terminal galactose residue. ST6Gal1 localizes on the cell membrane in the Golgi apparatus and in the extracellular region [11]. Similarly, α-2,3-ST I (ST3Gal1) catalyzes α-2,3 sialylation at sites such as those of ST6Gal1 and is involved in T-cell apoptosis and tumorigenesis [17,18,19]. Interestingly, ST6Gal1 modifies the sialylation of IgG Fc through α1-3 mannose [20]. Furthermore, ST activity intensifies in B cells in primary Sjögren’s syndrome [21]. Nonetheless, the only research on this subject to date is our exploration of the relationship between the monocyte ST6Gal1 and ST3Gal1 levels and disease activity in RA patients [22]. In addition, we found that the RA monocyte ST3Gal1 and Neu3 levels were correlated with DAS28-ESR scores greater than 5.1 [22]. Moreover, in a multi-variable analysis, the ST3Gal1 levels of RA monocytes were similarly associated with a DAS-ESR > 5.1, but this was not true for the CRP and the ESR [22]. These results (such as the demarcation of the enzyme levels between high and not-high RA activity) lead us to consider the possibility of using ST3Gal1 and Neu3 levels of RA monocytes to distinguish between RA activity improvement and remission. However, whether monocyte ST6Gal1 and ST3Gal1 levels can be used to discriminate the remission fulfillment and improvement criteria categories of RA patients is not yet known. Consequently, we attempted to examine the ST6Gal1 and ST3Gal1 levels to evaluate their role for their above-mentioned usage.

It is established that Neu desialylates SIA in cells. Neu1 is required throughout early IL-4 production in T cells during contact with antigen-presenting cells [23], the regulation of macrophage phagocytosis [24], and the production of IgG1 and IgE by B cells [25]. Neu1 exists on the cell surface and in the lysosomal compartment, whereas Neu3 is present only on the cell surface [26]. Neu3 is a ganglioside-specific sialidase and is involved in the modulation of cell-surface gangliosides [21]. In brief, the ST6Gal1/Neu1 pair operates along the α-2,6-SIA pathway, and the ST3Gal1/Neu3 pair acts in the α-2,3-SIA pathway. In particular, the presence and absence of α-2,6-SIA confer anti- and pro-inflammatory properties to IgG autoantibodies, respectively [27]. In turn, the sialylated IgG and the desialylated IgG connects with ST6Gal1 (to add α-2,6-SIA) and Neu1 (to remove α-2,6-SIA) in B cells, respectively; this further stresses the importance of these enzymes in RA pathophysiology [13,14].

We reported earlier that both the ST3Gal1 and Neu3 levels in the B cells of RA patients correlated positively with RA disease activity (DAS28-ESR) [28]. Moreover, the B-cell Neu3 levels were significantly associated with combined moderate and high RA disease activity (DAS28-ESR > 3.2) and with high RA disease activity (DAS28-ESR > 5.1) [28]. However, whether the B-cell or monocyte Neu1 and Neu3 levels of RA patients can discriminate remission definitions and improvement criteria in RA patients is currently unknown. Hence, through the same reasoning outlined above (concerning monocytes), we decided to examine the Neu1 and Neu3 levels together with the ST6Gal1 and ST3Gal1 levels of monocytes and B cells to determine whether they could act as markers for those usages and to understand different enzymes’ involvement in separate improvement categories. In contrast, because T cells and polymorphonuclear cells had no such correlation [22,28], they were not studied further. The aim of this study is to explore whether the ST6Gal1, Neu1, ST3Gal1, and Neu3 levels in the B cells and monocytes of RA patients could be useful for determining RA disease activity improvement and remission.

## 2. Results

We collected data from 100 RA patients, with a total of 312 visits (Table 1). The female-to-male ratio was 80:20. These RA patients had high SJCs, TJCs, and DAS28-ESR scores (Table 1). The low (in remission) DAS28-ESR score is less than 2.6 and the high DAS28-ESR score is above 5.1 [9].

### 2.1. AUC of B-Cell and Monocyte ST and Neu Levels and Ratios against Two Remission Definitions

The B-cell Neu1 and ST6Gal1 levels and monocyte ST6Gal1/Neu1 ratios along with the other B-cell enzymes by way of the receiver operating characteristic (ROC) curve could predict 2005 modified American Rheumatism Association (ARA) remission [29] and 2011 American College of Rheumatology/European League Against Rheumatism (ACR/EULAR) remission [30] (Table 2), unlike some other monocyte enzyme levels and ratios (all with AUCs < 0.600). B-cell Neu 1, cut off at 95.6 (mean fluorescence level), gave a sensitivity of 0.656 and a specificity of 0.669 for modified ARA remission and a sensitivity of 0.676 and a specificity of 0.678 for 2011 ACR/EULAR remission. According to the two remission definitions (Table 2), the performances of the B-cell Neu1 levels and other B-cell enzymes were acceptable; however, no meaningful results were observed for the blood CRP and ESR against modified ARA remission and 2011 ACR/EULAR remission (Table 2).

### 2.2. Treatment Responses Using Different Response (Improvement) Criteria

**Figure 1:**
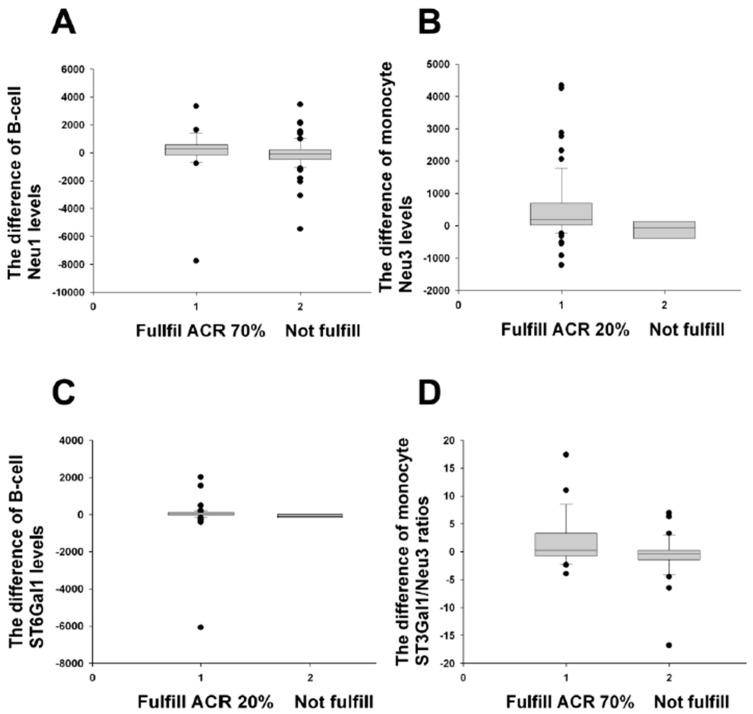
Comparisons of the difference of monocyte and B-cell enzyme levels/ratios between various ACR improvements and their non-improvements. (**A**) The B-cell Neu1 difference (M0 − M3) fulfilled the ACR 70% improvement criteria (*n* = 23), compared with those not fulfilled (*n* = 67), *p* = 0.043. (**B**) The monocyte Neu3 difference (M0 − M12) fulfilled the ACR 20% improvement criteria (*n* = 65), compared with those not fulfilled (*n* = 6), *p* = 0.037. (**C**) The B-cell ST6 difference (M0 − M12) fulfilled the ACR 20% improvement criteria (*n* = 62), compared with those not fulfilled (*n* = 5), *p* = 0.050. (**D**) The monocyte ST3/Neu3 ratio difference (M0 − M12) fulfilled the ACR 70% improvement criteria (*n* = 26), compared with those not fulfilled (*n* = 38), *p* = 0.042. All comparisons were analysed using the Mann–Whitney U test.

When ACR improvement [31] was calculated in this study, the difference in the B-cell Neu1 levels (that is, the enzyme levels at Month 0, upon enrollment, minus those at Month 3) demonstrated a significant disparity between the ACR 70% improvement group and the non-improvement group (Figure 1A). A similar distinction was also seen for the difference in the monocyte Neu3 and B-cell ST6Gal1 levels as well as the monocyte ST3Gal1/Neu3 ratios, which discriminated between the ACR improvement and the non-improvement groups (the enzyme levels at Month 0 minus those at Month 12) (Figure 1B–D). The changes in all other enzyme levels and ratios showed no difference between the ACR improvement group and the non-fulfilled group (all *ps* > 0.05). Because no difference between the improvement group and the non-improvement group was observed during M0 − M6, no M0 − M6 results are shown.

In contrast, only one positive result (B-cell Neu1) was obtained when we used an end-result DAS28-ESR < 3.2 with decreased scores of more than 1.2 as a EULAR (major) improvement criterion [32] (Table 3). These results differed from those in Figure 1, and the only identical biomarker was the B-cell Neu1 level. When the Simplified Disease Activity Index’s (SDAI) 50%, 70%, and 85% criteria [33] were applied (Table 4), obvious differences were noted in the comparisons (Table 4), contrary to the EULAR improvement group vs. the non-improvement group (Table 3). Hence, the B-cell Neu1 level may be used clinically owing to its accordance with three different improvement criteria (Figure 1, Table 3 and Table 4). The fulfillment of two improvement criteria (the ACR and SDAI) was seen for the monocyte Neu3 and B-cell ST6Gal1 levels and monocyte ST3Gal1/Neu3 ratios (Figure 1 and Table 4).

Whether such treatment responses also existed in the RA patients with positive autoantibodies or using biologics was further examined below.

### 2.3. Treatment Responses in RA Patients with Positive Rheumatoid Factor

**Figure 2:**
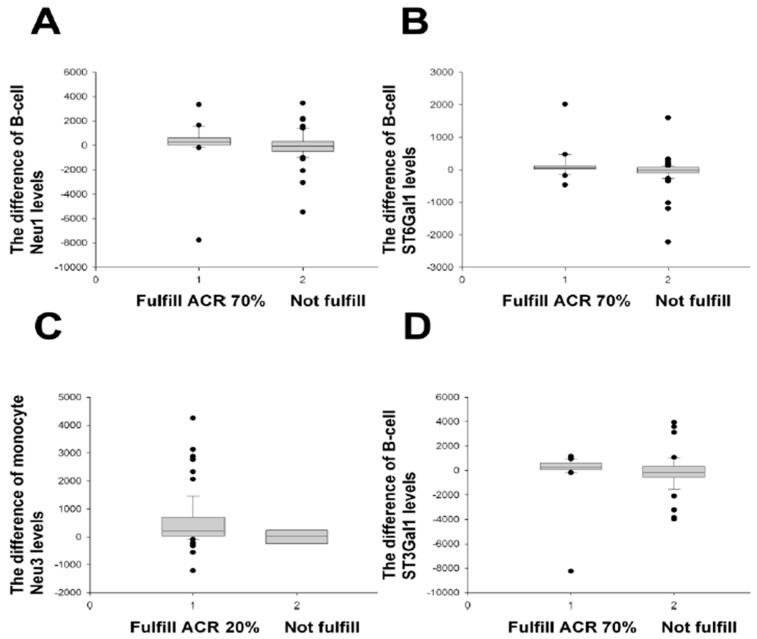
Comparisons of the difference of monocyte and B-cell enzyme levels/ratios between various ACR improvements and their non-improvements in RA patients with positive rheumatoid factor. (**A**) The B-cell Neu1 difference (M0 − M3) fulfilled the ACR 70% improvement criteria (*n* = 21), compared with those not fulfilled (*n* = 53), *p* = 0.041. (**B**) The B-cell ST6 difference (M0 − M3) fulfilled the ACR 70% improvement criteria (*n* = 21), compared with those not fulfilled (*n* = 50), *p* = 0.022; that of the B-cell ST6 difference yielded *p* = 0.024 (ACR 50%, M0 − M12). (**C**) The monocyte Neu3 difference (M0 − M12) fulfilled the ACR 20% improvement criteria (*n* = 67), compared with those not fulfilled (*n* = 8), *p* = 0.029; that of the monocyte ST6/Neu1 ratio difference rendered *p* = 0.007 (ACR 70%, M0 − M12); that of the monocyte ST6 difference yielded *p* = 0.019 (ACR 50%, M0 − M15). (**D**) The B-cell ST3 difference (M0 − M3) fulfilled the ACR 70% improvement criteria (*n* = 21), compared with those not fulfilled (*n* = 49), *p* = 0.013; that of the B-cell ST3 difference provided *p* = 0.046 (ACR 50%, M0 − M12); that of the B-cell Neu 3 difference yielded *p* = 0.015 (ACR 70%, M0 − M3). All comparisons were analysed using the Mann–Whitney U test.

When RA patients at Month 0 were classified by a positive rheumatoid factor (RF), the difference in the B-cell Neu1 levels was significantly disparate between the ACR 70% improvement group and the non-improvement group (Figure 2). A similar discrimination was also seen in the difference between the B-cell ST6Gal1 and monocyte Neu3 levels, which distinguished the ACR improvement group from the non-improvement group, similar to RA patients as a whole as in Figure 1 (Figure 2). Additionally, the difference in the B-cell ST3Gal1 (Figure 2) and B-cell Neu3 (*p* = 0.015) levels was good for differentiating ACR improvement (items showing a higher patient number in remission were shown).

Three positive results (B-cell Neu1, B-cell ST3Gal1, and B-cell Neu3) were obtained to fulfill the major EULAR improvement criteria (Table 5).

When the SDAIs 50%, 70%, and 85% criteria were applied to the positive RF group (Supplementary Table S1), the inclusion of more positive items was noted in the comparisons, contrary to the ACR (Figure 2) and EULAR (Table 5) improvement groups vs. the non-improvement groups.

Altogether, the B-cell Neu1 levels classified by a positive RF were superior for discrimination according to the three different improvement criteria, similar to RA patients as a whole (Figure 1, Table 3 and Table 4).

### 2.4. Treatment Responses in RA Patients with Positive Anti-Cyclic Citrullinated Peptide (Anti-CCP) Antibodies

**Figure 3:**
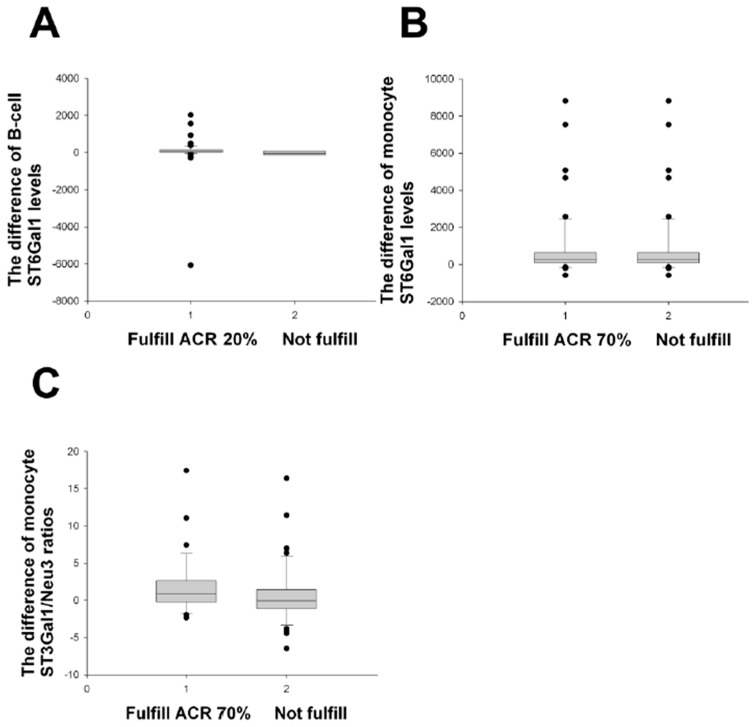
Comparisons of the difference of monocyte and B-cell enzyme levels/ratios between various ACR improvements and their non-improvements in RA patients with positive anti-CCP antibodies. (**A**) The B-cell ST6 difference (M0 − M12) fulfilled the ACR 70% improvement (*n* = 75) criteria, compared with those not fulfilled (*n* = 6), *p* = 0.030. (**B**) The monocyte ST6 difference gave *p* = 0.039 for ACR 70% improvement (*n* = 50) vs. non-improvement (*n* = 31) (M0 − M15). (**C**) The monocyte ST3/Neu3 ratio difference rendered *p* = 0.036 for ACR 70% improvement (*n* = 33) vs. non-improvement (*n* = 41) (M0 − M12). All comparisons were analysed using the Mann–Whitney U test.

When the RA patients at Month 0 were classified by positive anti-CCP antibodies, the B-cell Neu1 levels of the ACR improvement group and the non-improvement group (all *p* > 0.05) were not different, dissimilar to results in Figure 1 and Figure 2. Nevertheless, the difference in the B-cell ST6Gal1 and the monocyte ST3Gal1/Neu3 ratios significantly distinguished the ACR improvement group from the non-improvement group (Figure 3), similar to results in Figure 1.

Only one positive result concerning the difference in the B-cell Neu1 levels was acquired that fulfilled the major EULAR improvement criteria (Table 6), similar to results in Table 3.

When the SDAIs 50%, 70%, and 85% criteria were applied to the positive anti-CCP group (Supplementary Table S2), the inclusion of more positive items was noted in the comparisons, contrary to the ACR (Figure 3) and EULAR improvement groups vs. non-improvement groups (Table 6).

Altogether, the B-cell Neu1 levels classified by a positive anti-CCP were good for the discrimination of two different improvement criteria (Table 6 and Supplementary Table S2).

### 2.5. Treatment Responses in RA Patients with the Use of Biologics

**Figure 4:**
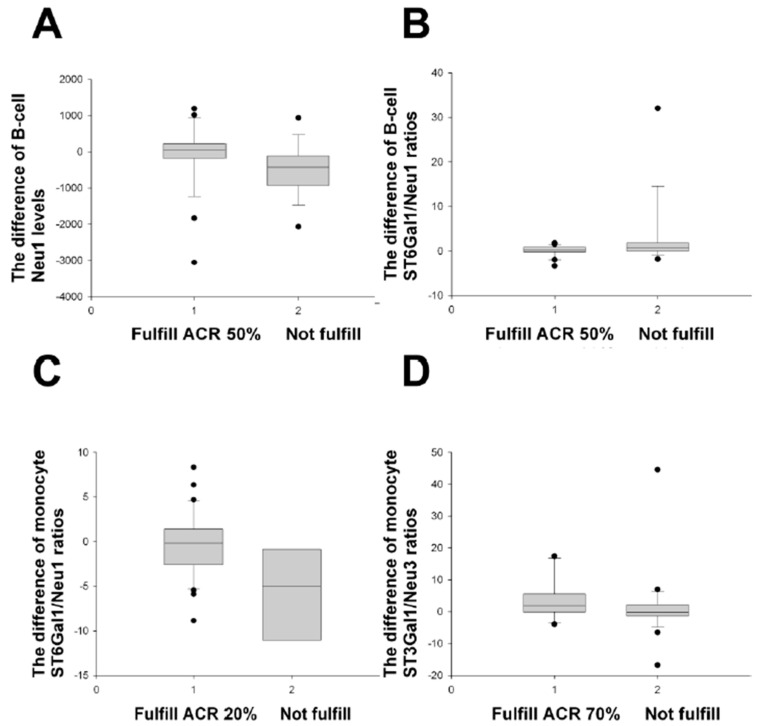
Comparisons of the difference of monocyte and B-cell enzyme levels/ratios between various ACR improvements and their non-improvements in RA patients with use of biologics. (**A**) The B-cell Neu1 difference (M0 − M3) fulfilled the ACR 50% improvement criteria (*n* = 26), compared with those not fulfilled (*n* = 16), *p* = 0.029. (**B**) The B-cell ST6/Neu1 ratio difference (M0 − M3) fulfilled the ACR 50% improvement (*n* = 21), compared with those not fulfilled (*n* = 15) gave *p* = 0.037. (**C**) The monocyte ST6/Neu1 ratio difference (M0 − M3) fulfilled the ACR 20% improvement (*n* = 31), compared with those not fulfilled (*n* = 6), *p* = 0.009. (**D**) The monocyte ST3/Neu3 ratio difference (M0 − M12) fulfilled the ACR 70% improvement (*n* = 10), compared with those not fulfilled (*n* = 27), *p* = 0.028. All comparisons were analysed using the Mann–Whitney U test.

When RA patients at Month 0 were classified by use of biologics, the difference in the B-cell Neu1 levels was significantly dissimilar between the ACR improvement group and the non-improvement group (Figure 4), the same as the results in Figure 1 and Figure 2, but different from those in Figure 3. The same results were found for the difference in the monocyte ST3Gal1/Neu 3 ratios. However, the difference in the B-cell ST6Gal1/Neu1 ratios and monocyte ST6Gal1/Neu1 ratios significantly distinguished the ACR improvement group from the non-improvement group (Figure 4), different from the results in Figure 1, Figure 2 and Figure 3.

Firstly, RA patients using biologics were selected. The value 0.028 in Table 7 is a *p*-value for the difference in B-cell Neu1 levels (M0 − M12) that was compared between the EULAR improvement group and the non-improvement group (the improvement was based on the end-result DAS28-ESR < 3.2 (M12) and decreased scores of more than 1.2 (the DAS28-ESR value at Month 0 minus the DAS28-ESR value at Month 12). Again, only one positive result, the difference in the B-cell Neu1 levels, was acquired that fulfilled the major EULAR improvement criteria for the group using biologics (Table 7), similar to results in Table 3 and Table 6.

When the SDAIs 50%, 70%, and 85% criteria were applied to the group using biologics (Supplementary Table S3), the inclusion of several positive items was noted in the comparisons, similar to the ACR improvement group vs. non-improvement group (Figure 4) and contrary to results in Table 7.

Altogether, the B-cell Neu1 levels classified by the use of biologics were superior for the discrimination of three different improvement criteria (Figure 4, Table 7 and Supplementary Table S3), similar to RA patients as a whole (Figure 1, Table 3 and Table 4).

### 2.6. Comparison of Enzyme Levels between Different Categories of DAS28-ESR and DAS28-MCP-1 Scores

The B-cell and monocyte ST3Gal1, Neu3, ST6Gal1 and Neu3 levels were higher in DAS28-ESR non-remission (≥2.6) than those in DAS28-ESR remission (<2.6) (Supplementary Figures S1A–D and S2A–D). The B-cell and monocyte ST3Gal1/Neu3 and ST6Gal1/Neu3 ratios were higher in DAS28-ESR remission (<2.6) than those in DAS28-ESR non-remission (≥2.6) (Supplementary Figures S1E,F and S2E,F). The graphs with differences between the groups that were hard to clearly visualize (Supplementary Figures S1C–F and S2E) were given their 25th percentile, median, and 75th percentile data in Supplementary Table S4.

The B-cell and monocyte ST3Gal1, Neu3, ST6Gal1, and Neu3 levels were higher in DAS28-MCP-1 non-remission (≥2.2) than those in DAS28-MCP-1 remission (<2.2) [28] (derived from the data in Ref. [28]) (Supplementary Figures S3A–D and S4A–D). The B-cell and monocyte ST3Gal1/Neu3 and ST6Gal1/Neu3 ratios were not different in DAS28-MCP-1 remission (<2.2) from those in DAS28-MCP-1 non-remission (≥2.2) (Supplementary Figures S3E,F and S4E,F), except that the monocyte ST6Gal1/Neu1 ratios were higher with a DAS28-MCP-1 < 2.2 (higher) and a DAS-MCP ≥ 2.2 (Supplementary Figure S4F). The graphs with differences between the groups that were hard to clearly visualize (Supplementary Figures S3C,E,F, and S4C,E) were given their 25th percentile, median, and 75th percentile data in Supplementary Table S5.

## 3. Discussion

The disease activity measures for RA, such as the DAS28-ESR, have been validated and widely used [34]. However, the ESRs engaged when calculating the DAS28 are in normal ranges for up to 40% of RA patients [35,36]. Thus, other biomarkers relating to the disease activity measure of RA are extensively sought after [37,38,39,40]. This is because biomarkers offer objective assessments of RA disease activity beyond evaluating the SJC, TJC, and general health (GH).

Earlier, the EULAR improvement criteria (response criteria) were seen to display good discrimination validity, but this was not the case for ACR 20% improvement (the 20% response criteria) [32]. Later, it was reported that the EULAR improvement criteria had a validity comparable to the ACR improvement criteria [41]. Nevertheless, in that report [41], the US trials had five out of seven trials possessing chi-squared ratios above or below 1, including two trials’ chi-squared ratios of 1.8. This indicates that the ACR improvement criteria have discriminant validity that is different from the EULAR improvement criteria. These results do match with our results in terms of the B-cell and monocyte ST and Neu enzymes and related ratios performing different discriminant abilities for the ACR and EULAR improvement group vs. the non-improvement group (Figure 1 and Table 3) except for one common biomarker, the B-cell Neu1 level. The B-cell Neu1 level’s discriminative capability for fulfilling all three of the ACR, EULAR, and SDAI responses is quite likely related to Neu1′s extended ability to affect T and B cells and macrophages [23,24,25].

Similarly, the B-cell and monocyte ST3Gal1, Beu3, ST6Gal1, and Neu1 levels, ST3Gal1/Neu3 ratios, and St6Gal1/Neu1 ratios can be used to discriminate DAS28-ESR remission from non-remission (Supplementary Figures S1 and S2). Again, the B-cell and monocyte ST3Gal1, Beu3, ST6Gal1, and Neu1 levels can be used to distinguish DAS28-MCP-1 remission from non-remission (Supplementary Figures S3 and S4A–D). Nevertheless, only the monocyte ST6Gal1/Neu1 ratios can differentiate a DAS-MCP-1 < 2.2 from a DAS-MCP ≥ 2.2 (Supplementary Figure S4F).

**Figure 5:**
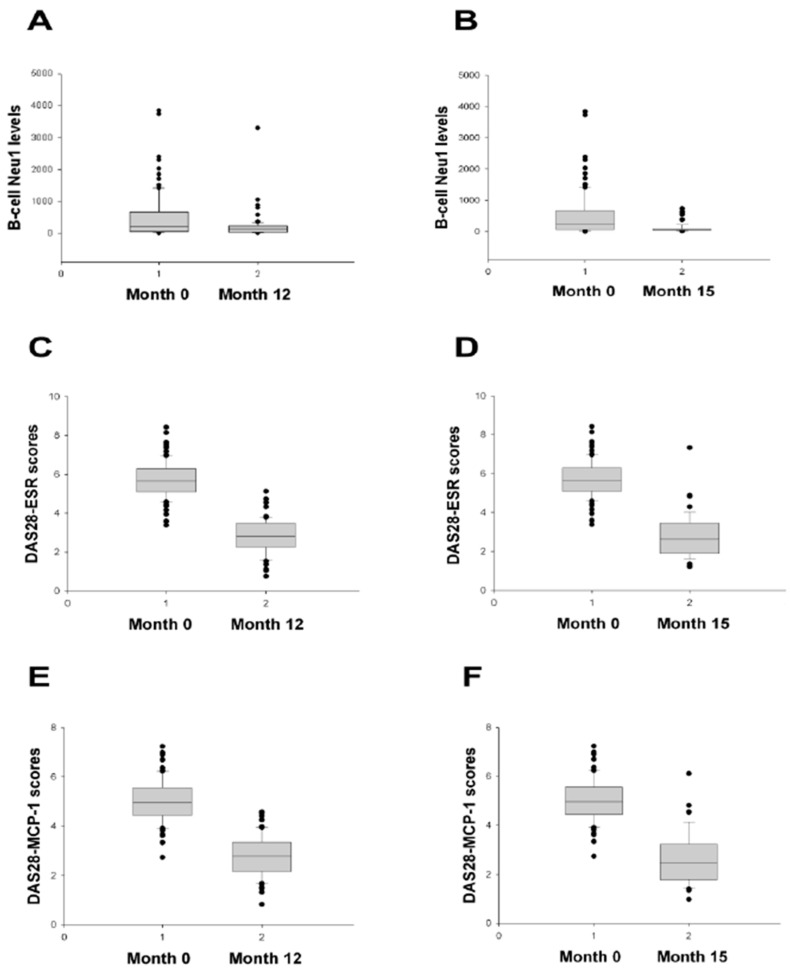
Comparison of the B-cell Neu1 level and DAS28 scores between baseline and later months. The B-cell Neu1 level was significantly lower in (**A**) month 12 (*n* = 72) and (**B**) month 15 (*n* = 48) than month 0 (*n* = 100) (*p* = 0.009 and <0.001, respectively). DAS28-ESR scores were significantly lower in (**C**) month 12 and (**D**) month 15 than month 0 (*p* < 0.001 and <0.001, respectively). DAS28-MCP-1 scores were significantly lower in (**E**) month 12 and (**F**) month 15 than month 0 (*p* < 0.001 and <0.001, respectively). All comparisons were analysed using the Mann–Whitney U test.

The B-cell Neu1 level being lower in Month 12 and Month 15 than in Month 0 (Figure 5A,B) indicated its lower ability to deplete the α-2,6-SIA of the IgG secreted by B-cells. That is, low B-cell Neu1 levels were attuned with a lower DAS28-ESR and a lower DAS28-MCP-1 [8] in Month 12 and Month 15 than in Month 0 (Figure 5C–F). A similar compatibility was seen for the DAS28-CRP and SDAI [8] (all *p*-values < 0.001). Then, the reason why the B-cell ST6Gal1 level (which can increase the α-2,6-SIA of the IgG secreted by B-cells) and the B-cell ST6Gal1/Neu1 ratio (for which higher ratios can increase the α-2,6-SIA of the IgG secreted by B-cells) did not show such compatibility (Figure 5: not shown), seems to relate to the extracellular existence of ST6Gal1 in addition to that found on the cell membrane and in the Golgi apparatus [16]. In contrast, Neu1 does not exist in the extracellular region and exists only on and inside the cells [26]. Moreover, the low B-cell Neu1 level in Month 12 and Month 15 (low DAS28 scores) is compatible with the higher SIA/anti-CCP ratios in the DAS28-ESR < 2.6 remission group and the higher IgG α-2,6-SIA/IgG ratios in the low DAS28-ESR patients [42]. Therefore, higher α-2,6-SIA in the autoantibodies secreted by B-cells is associated with lower DAS28 scores and lower B-cell Neu1 levels after 12-month therapy and autoantibodies become more anti-inflammatory as described earlier [27,43,44].

The role of α-2,6-sialylated IgG in joint inflammation was firstly demonstrated in mice by Kaneko, Nimmerjahn, and Ravetch in 2006 [27]. The study suggested that neuramindase-treated (to remove α-2,6 SIA from IgG) intravenous immunoglobulin G (IVIg) did not retain the anti-inflammatory effect of IVIg in K/BxN-serum-induced arthritis in mice. In contrast, IVIG with the enrichment of α-2,6-sialic acid obtained from a Sambucus nira agglutin (SNA)-lectin affinity column, not only retained the anti-inflammatory effect on the arthritis score of IVIg, but also significantly suppressed neutrophil infiltration markedly in the synovium of K/BxN-serum treated mice [27].

The above results of ours suggest that B-cell ST6Gal1 and Neu1 probably involve in the pathophysiology of disease remission in RA through modulation of the α-2,6 SIA content in IgG (or IgG autoantibodies). Similarly, B-cell ST3Gal1 and Neu3 likewise meaningfully indicate/predict 2005 Modified ARA remission and 2011 ACR/EULAR remission (Table 2). It is intriguing, however, that the implication of α-2,3 SIA, which can be added onto IgG by B-cell ST3Gal1 and removed from IgG by B-cell Neu3 in the pathophysiology of disease remission in RA, has not yet been reported and future investigations must confirm.

In terms of improvement assessment in RA disease activity, we suggest that B-cell ST6Gal1, B-cell Neu1, monocyte Neu3, and monocyte ST3Gal1/Neu3 ratios are useful for differentiating ACR (Figure 1) and SDAI (Table 4) improvement vs. non-improvement; this further stresses their potential involvement through modulation of the α-2,6 SIA content in IgG (or IgG autoantibodies) in the pathophysiology of disease activity improvement in RA. Again, how monocyte Neu3 and ST3Gal1/Neu3 ratios achieve such improvement effects in RA is completely unknown and future research is much needed. To support this, we have reported earlier that monocyte Neu3 correlates with DAS28-ESR scores in RA patients [22].

Above all, B-cell Neu1 is the best to differentiate RA activity improvement jointly for ACR improvement (Figure 1), EULAR improvement (Table 3), and SDAI improvement (Table 4). The comparison of B-cell Neu1 with other enzymes can be seen in Table 8 and in subgroups (Table 9), further stressing the superiority of B-cell Neu1 among all enzymes studied in fulfilling two criteria systems for remissions (Table 2) and discriminating three different improvements.

Both the DAS28-ESR score and DAS28-CRP score have been commonly used as disease activity measurement tools for RA patients [9]. Aside from these, we added another useful disease activity formula, a DAS28-MCP-1 score, based upon our previously published studies for comparison [6,8]. The involved laboratory assay for these scores are the determinations of ESR, CRP, and MCP-1 that have been studied in a more detailed way (see below). It has been reported that serum CRP levels correlated significantly with the level of inflammation in the synovial tissue from RA patients (rho = 0.43 and *p* = 0.0001) [45]. Similarly, ESR levels correlated significantly with the level of synovial inflammation from RA patients (rho = 0.29 and *p* = 0.0001) [45]. Comparison of CRP and ESR levels across the 3-point synovial inflammation score yielded significant differences for CRP and ESR (*p* = 0.002 and 0.001, respectively, by the Kruskaii–Wallis test) [45]. Moreover, DAS28-CRP displayed a weak but significant correlation with synovial inflammation at rho = 0.23 and *p* = 0.0011. Nevertheless, the correlation between DAS28-ESR and synovial inflammation has not been reported [45].

Similarly, the synovial tissue was stained more intensely for MCP-1 in those with active inflammation than those with weak inflammation in RA patients [46]. Moreover, the MCP-1 level was significantly higher in the synovial fluid and the percentage of synovial macrophages for MCP-1 staining was significantly greater in RA patients than those in osteoarthritis patients [47]. Interestingly, an antagonist for MCP-1 obviated or decreased arthritis occurrence in MRL-lpr mice [48]. These study results suggest that MCP-1 plays an important role in RA pathophysiology.

A limitation of this study is that we had a very small number of visits in Figure 1, Figure 2 and Figure 3 for non-fulfilled ACR improvement: all the visits were from Month 0 minus Month 12. This condition implies our successful treatment of the RA patients after 12 months of therapy; thus, the situation of small numbers for non-fulfilled ACR improvement seems unavoidable.

## 4. Materials and Methods

### 4.1. Patient Enrollment

Patients who fulfilled the 1987 ACR criteria of RA and followed up for at least 3 months were recruited with written consent obtained from all patients. These patients were located at Chang Gung Memorial Hospital at both the Lin-kou branch and the Taipei branch and were under treatment with traditional or biological DMARDs for more than 3 months. We examined the monocyte and B-cell SIA-related enzyme (ST3Gal1, Neu3, ST6Gal1 and Neu1) levels of these patients according to the following categories over a 1-year and three-month period. The enrollment category included three periods: (1) when preparing to deliver the second DMARD to patients who had received one DMARD for ≥3 months; adjusting treatment every 1–3 months [49]; (2) when preparing to deliver a biological DMARD to patients who had received two DMARDs for ≥6 months with DAS28-ESR score > 5.1 (local Taiwan National Health Insurance (NHI) policy); and (3) when preparing to transition the patient to another biological DMARD after having received one kind of biological DMARD for ≥3 months (local Taiwan NHI policy). The third period allowed for a change in biological DMARDs every 3 months after the patients had received one kind of biological DMARD but had achieved unsatisfactory treatment effects (a decrease in the DAS28 score of no more than 1.2 compared with the previous score). The enrolled patients underwent blood sampling 5 times (Month 0 (M0), M3, M6, M12 and M15) based on the three collection criteria above every three months (see Reference [50] and based on local Taiwan National Health Insurance policy). The gap between M6 and M12 is to avoid too frequent blood withdrawal for enrolled RA patients’ willing to donate blood for research within the first year and later. The benefit of adding one biological DMARD [50] or changing into another biological DMARD [51] could relieve persistent painful joint swelling and subsequent disability. A total of 100 patients was enrolled, and 312 visits were recorded. The SJC, TJC, GH (same as the patient’s global assessment, PGA), evaluator’s global assessment (EGA) and Health Assessment Questionnaire Disability Index (HAQ-DI) were performed during each patient visit. The above plus laboratory data were used to calculate DAS28 scores based on the following formula: DAS28-ESR score = [0.56 × √TJC] + [0.28 × √SJC] + 0.70 × ln[ESR] + 0.014 × GH [in mm]; DAS28-CRP score {= [0.56 × √TJC] + [0.28 × √SJC] + (0.36 × ln[CRP; in mg/L]) + 1) + (0.014 × PGA [in mm]) + 0.96)}; SDAI {= SJC + TJC + PGA [VAS; in cm] + EGA [VAS; in cm] + CRP [in mg/dL])} and DAS28-MCP-1 score {= 0.56 × √TJC + 0.28 × √SJC + 0.39 × ln(MCP-1; in pg/mL) + 0.014 × (PGA [in mm])}. They were calculated as previously described [8].

### 4.2. Cell Staining and Flow Cytometric Detection

Peripheral blood mononuclear cells (PBMCs) were processed and obtained as previously described, and their monocyte and B-cell ST3Gal1, Neu3, ST6Gal1 and Neu1 levels were examined [22]. Firstly, blood from two 10-mL collection tubes containing ethylenediaminetetraacetic acid was centrifuged at 650× *g* (Kubota 2420, Osaka, Japan) for 10 min at 25 °C into layers of cells and plasma. After plasma was removed, cells were mixed with equal volume of phosphate buffered saline (PBS). The cell solution was then collected into 50 mL plastic tubes containing 10 mL Ficoll-Paque (Cytiva Europe, Uppsala, Sweden), which was followed by centrifuging at 800× *g* for 30 min at 25 °C to obtain interface cells as PBMCs as described with minor modifications [52]. Briefly, PBMCs in PBS were stained with phycoerythrin (PE)-conjugated mouse anti-human CD14 (clone: M5E2) (monocytes) and PE-conjugated mouse anti-human CD19 (clone: HIB19) (B cells) (BD Pharmingen, Mountain View, CA, USA). The isotype control was PE-conjugated mouse IgG1 *k* isotype control (clone: MOPC-21) (for both anti-CD14 and anti-CD19 staining) (BD Pharmingen, Mountain View, CA, USA). Moreover, rabbit polyclonal IgG anti-ST3Gal1 was added at 0.58 µg/mL, rabbit polyclonal IgG anti-Neu3 was added at 0.42 µg/mL, mouse monoclonal IgM anti-ST6Gal1 (clone: MOPC1041) was added at 0.36 µg/mL, or rabbit polyclonal IgG anti-Neu1 was added at 0.42 µg/mL (all from Abcam, Cambridge, MA, USA) for each test. Rabbit polyclonal IgG (Jackson ImmunoResearch, West Grove, PA, USA) was used at 2 µg/mL (Sigma, St. Louis, MO, USA) as the control for each test.

After being washed and centrifuged (Eppendorf 5810R, Hamburg, Germany: 644× *g* for 5 min), the cells were suspended in 100 µL of PBS; rabbit IgG was stained with allophycocyanin (APC)-goat antibody at 1 µg/mL (Abcam, Cambridge, MA, USA), and mouse IgM was stained with APC-monoclonal rat antibody (1B4B1) at 1 µg/mL (MyBioSource, San Diego, CA, USA) for each test. Afterwards, the cells were washed and resuspended in 500 µL of PBS (4 °C) for analysis by using the BD FACSCalibur System (BD Biosciences, San Jose, CA, USA). The smallest number of cells collected for analysis was 20,000, and all flow cytometric data were analyzed using the FlowJo 7.6.1 program (FlowJo, LLC, Ashland, OR, USA).

The geometric mean fluorescence intensity for staining of each cell depended on the enzyme level in the cell. The examined parameters were CD14, CD19, ST3Gal1, Neu3, ST6Gal1 and Neu1.

Both monocytes and B cells were stained for the same four enzymes:

(a) ST3Gal1: rabbit polyclonal IgG anti-ST3Gal1 (control: rabbit IgG) plus allophycocyanin (APC)-goat antibody against rabbit IgG
(b) Neu3: rabbit polyclonal IgG anti-Neu3 (control: rabbit IgG) plus APC-goat antibody against rabbit IgG
(c) ST6Gal1: mouse monoclonal IgM anti-ST6Gal1 (control: mouse IgM) plus APC-monoclonal rat antibody against mouse IgM
(d) Neu1: rabbit polyclonal IgG anti-Neu1 (control: rabbit IgG) plus APC-goat antibody against rabbit IgG

### 4.3. Determination of Rheumatoid Factor and Anti-Cyclic Citrullinated Peptide Antibodies

At baseline, serum IgM RF (measured through nephelometry with N Latex RF Kit from Siemens Healthcare Diagnostics Products GmbH, Marburg, Germany) and anti-CCP antibodies (Quanta Flash CCP3 Reagents; Inova Diagnostics, Inc., San Diego, CA, USA) were examined.

Methods for determining RF and anti-CCP antibodies are as follows: (a) RF determination was carried out by our Hospital Laboratory Medicine Department (clinical laboratory) based on the method of Nephelometry: 3 mL of blood was collected into a Greiner Bio-One (Germany) Vancutte tube (containing Z serum separator clot activator) and centrifuged at 1880× *g* for 10 min at 25 °C with the centrifuge Kubota 8410 (Tokyo, Japan) to obtain at least 1 mL of serum. The serum was then placed into the SIEMENS BN Prospec machine (SIEMENS, Oststeinbek, Germany) to react with particles containing human IgG/sheep anti-human IgG immune complexes. After being projected by the machine light beam, the scattered light of the reaction particles was detected and plotted against the standard curve supplied by the manufacturer (Instruction Manual for BN Prospec System 2005, SIEMENS, Oststeinbek, Germany) to obtain international units (IU)/mL for RF. (b) Determination of anti-CCP antibodies was carried out by our Hospital Clinical Immunology Laboratory based on the chemiluminescence assay supplied by Inova Inc. Sera with more than 300 μL were obtained from blood collected in a Greiner Bio-One tube (Greiner Bio-One GmbH, Frickenhausen, Germany). Then, the sera were placed into tubes inside the BIO-FLASH machine (Inova, Inc., San Diego, CA, USA) and processed according to the procedures set out by the manufacturer (QUANTA Flash CCP3 Reagents, Inova Diagnostics, Inc., San Diego, CA, USA).

RF may form immune complexes with IgG-containing antigen-antibody complexes to induce complement activation and subsequent inflammation inside the joint [53]. Moreover, bone, and cartilage damage occur more likely in RA patients with positive anti-CCP antibodies [54].

### 4.4. Statistical Analyses

The AUC under the ROC curve of different variables of B-cell and monocyte ST and Neu levels and ratios as well as blood CRP and ESR against two remission definitions was calculated [29,30]. The difference in the B-cell and monocyte enzyme levels from different months between improvement-fulfilling and non-improvement subgroups [31,32,33] was compared through Mann–Whitney U test. Any *p*-values less than 0.05 (two-tailed) were considered significant.

## 5. Conclusions

In summary, we have pioneered an approach to establish novel biomarkers for discriminating the ACR/EULAR/SDAI improvement from the non-improvement. This study is the first to connect the cell-surface SIA-related enzyme levels of B cells and monocytes with the ability to distinguish the improvement vs. the non-improvement of the three presently available improvement criteria [31,32,33]. In particular, and practically important, the B-cell Neu1 level is prospectively useful to distinguish all three ACR, EULAR, and SDAI improvements followed by the usefulness of the B-cell ST6Gal1 and monocyte Neu3 levels and monocyte ST3Gal1/Neu3 ratios to discriminate both ACR and SDAI improvements. These results might imply different immunologic abnormalities behind the sialic-acid-related enzymes of B cells and monocytes in ACR, EULAR and SDAI improvements. The immunologic basis behind these findings can only be deciphered through future studies.

## Figures and Tables

**Table 1:** Demographic, clinical, and laboratory data of patients with rheumatoid arthritis.

	Median (or Mean ± SD)	Quartiles (25–75%) or Range
Patient number (female: male)	100 (80:20)	
Total visits	312	
Age	55.4 ± 13.0	22–79
Disease duration (months from initial arthritic symptoms)	58.5	5.0–114.8
Swollen joint count	6.5	5.0–10.0
Tender joint count	8.0	5.0–12.5
Global health	80	50–80
Morning stiffness (min)	60	22.5–172.5
ESR (mm/h)	28.0	17.0–44.8
Rheumatoid factor (IU/mL)	59.9	15.2–296.5
Anti-CCP (mg/mL)	279.8	50.8–336.9
DAS28-ESR	5.7 ± 1.0	3.4–8.4
HAQ-DI	1.00	0.00–3.33

Anti-CCP represents IgG anti-cyclic citrullinated peptide antibody, DAS28-ESR represents Disease Activity Score 28 with inclusion of ESR, and HAQ-DI represents Health Assessment Questionnaire Disability Index. The low and high limits: swollen joint count (0–28) and tender joint count (0–28). Normal ranges: ESR < 15 mm/h, rheumatoid factor < 15 IU/mL, and anti-CCP < 20 mg/mL.

**Table 2:** Areas under the curve for monocyte and B-cell enzyme levels and ratios, CRP, and ESR against two remission definitions.

	Area under the Curve	Asymptotic Significance	Asymptotic 95% Confidence Interval
Modified ARA remission			
Monocyte ST6/Neu1 ratios	0.634	*p* = 0.013	0.532–0.736
B-cell ST3Gal1	0.658	*p* = 0.003	0.568–0.749
B-cell Neu3	0.641	*p* = 0.009	0.549–0.734
B-cell ST6Gal1	0.648	*p* = 0.006	0.554–0.742
B-cell Neu1	0.674	*p* = 0.001	0.538–0.764
CRP (mg/L)	0.331	*p* = 0.002	0.256–0.406
ESR (mm/h)	0.356	*p* = 0.004	0.283–0.428
2011 ACR/EULAR remission			
Monocyte ST6/Neu1 ratios	0.672	*p* = 0.001	0.583–0.762
B-cell ST3Gal1	0.633	*p* = 0.009	0.537–0.729
B-cell Neu3	0.662	*p* = 0.001	0.572–0.751
B-cell Neu1	0.682	*p* < 0.001	0.593–0.772
CRP (mg/L)	0.368	*p* = 0.014	0.290–0.445
ESR (mm/h)	0.416	*p* = 0.097	0.330–0.502

Modified ARA remission represents 2005 modified American Rheumatism Association remission [29]; ST3 represents α-2,3-sialyltransferase I; Neu3 represents neuraminidase 3; ST6 represents α-2,6-sialyltransferase I Neu1 represents neuraminidase 1; ST6/Neu1 represents α-2,6-sialyltransferase I/neuraminidase 1 ratios; and 2011 ACR/EULAR remission represents 2011 American College of Rheumatology/European League Against Rheumatism remission [30].

**Table 3:** Comparisons of the difference in B-cell and monocyte enzyme levels and ratios between the EULAR improvement group and the non-improvement group.

	B-Cell ST3	B-Cell Neu3	B-Cell ST6	B-Cell Neu1	B-Cell ST3/Neu3	B-Cell ST6/Neu1
M0 − M3	-	-	-	-	-	-
M0 − M12	-	-	-	0.014	-	-
M0 − M15	-	-	-	-	-	-

EULAR represents European League Against Rheumatism. *p*-values are shown; “-” indicates *p*-values > 0.05. ST3 represents α-2,3-sialyltransferase I; Neu3 represents neuraminidase 3; ST6 represents α-2,6-sialyltransferase I; Neu1 represents neuraminidase 1; ST6/Neu1 represents α-2,6-sialyltransferase I/neuraminidase 1 ratios; and ST3/Neu3 represents α-2,3-sialyltransferase I/neuraminidase 3 ratios. The EULAR improvement was calculated with the end-result DAS28-ESR < 3.2 and decreased scores of more than 1.2 among groups of M0 minus M3 (M0 − M3), M0 minus M12 (M0 − M12) and M0 minus M15 (M0 − M15). The non-improvement refers to those with the end-result DAS28-ESR > 3.2 and/or decreased scores of less than 1.2. Then, the difference in separate enzyme levels and ratios between fulfillment and non-fulfillment groups was compared to obtain *p*-values. All comparisons were performed through the Mann–Whitney U test. Other monocyte results were all negative.

**Table 4:** Comparing the difference in B-cell and monocyte enzyme levels and ratios between the SDAI improvement group and the non-improvement group.

	B-Cell ST3	B-Cell Neu3	B-Cell ST6	B-Cell Neu1	Mono ST3	Mono Neu3	Mono ST6	Mono Neu1	B-Cell ST3/Neu3	B-Cell ST6/Neu1	Mono ST3/Neu3	Mono ST6/Neu1
M0 − M3	-	0.047 ^#^	-	0.018 ^#^	-	-	-	-	-	-	-	-
M0 − M12	0.003 *	0.013 *; 0.020 ^#^	0.007*	0.001 *; 0.035 ^#^	0.028 ^@^	0.001 *	0.023 *	<0.001 *	0.014 *	-	0.044 ^@^	0.007 ^@^
M0 − M15	-	-	-	-	-	-	-	-	-	-	-	-

SDAI represents Simplified Disease Activity Index. *p*-values are shown; “-” indicates *p*-values > 0.05. ST3 represents α-2,3-sialyltransferase I; Neu3 represents neuraminidase 3; ST6 represents α-2,6-sialyltransferase I; Neu1 represents neuraminidase 1; ST6/Neu1 represents alpha-2,6-sialyltransferase I/neuraminidase 1 ratios; ST3/Neu3 represents α-2,3-sialyltransferase I/neuraminidase 3 ratios; and Mono represents monocyte. The SDAI improvement was calculated with 50% *, 70% ^#^ and 85% ^@^ reduction among groups of M0 minus M3 (M0 − M3), M0 minus M12 (M0 − M12) and M0 minus M15 (M0 − M15). The non-improvement refers to those with less than 50%, 70% and 85% reduction. Then, the difference in separate enzyme levels and ratios between the fulfillment and the non-fulfillment groups was compared to obtain *p*-values. All comparisons were performed through the Mann–Whitney U test.

**Table 5:** Comparisons of the difference in B-cell and monocyte enzyme levels and ratios between the EULAR improvement group and the non-improvement group of RA patients with positive rheumatoid factor.

	B-Cell ST3	B-Cell Neu3	B-Cell ST6	B-Cell Neu1	B-Cell ST3/Neu3	B-Cell ST6/Neu1
M0 − M3	-	-	-	-	-	-
M0 − M12	0.016	0.016	-	0.016	-	-
M0 − M15	-	-	-	-	-	-

*p*-values are shown. “-” indicates *p*-values > 0.05. ST3: α-2,3-sialyltransferase I; Neu3: neuraminidase 3; ST6: alpha-2,6-sialyltransferase I; Neu1: neuraminidase 1; ST3/Neu3: α-2,3-sialyltransferase I/neuraminidase 3 ratios; and ST6/Neu1: α-2,6-sialyltransferase I/neuraminidase 1 ratios. The EULAR improvement was calculated with the end-result DAS28-ESR < 3.2 and decreased scores of more than 1.2 among groups of M0 minus M3 (M0 − M3), M0 minus M12 (M0 − M12) and M0 minus M15 (M0 − M15).

**Table 6:** Comparisons of the difference in B-cell and monocyte enzyme levels and ratios between the EULAR improvement group and the non-improvement group of RA patients with positive anti-CCP antibodies.

	B-Cell ST3	B-Cell Neu3	B-Cell ST6	B-Cell Neu1	B-Cell ST3/Neu3	B-Cell ST6/Neu1
M0 − M3	-	-	-	-	-	-
M0 − M12	-	-	-	0.037	-	-
M0 − M15	-	-	-	-	-	-

*p*-values are shown; “-” indicates *p*-values > 0.05. ST3: α-2,3-sialyltransferase I; Neu3: neuraminidase 3; ST6: α-2,6-sialyltransferase I; Neu1: neuraminidase 1; ST3/Neu3: α-2,3-sialyltransferase I/neuraminidase 3 ratios; and ST6/Neu1: α-2,6-sialyltransferase I/neuraminidase 1 ratios. The EULAR improvement was calculated with end-result DAS28-ESR < 3.2 and decreased scores of more than 1.2 among groups of M0 minus M3 (M0 − M3), M0 minus M12 (M0 − M12) and M0 minus M15 (M0 − M15).

**Table 7:** Comparisons of the difference in B-cell and monocyte enzyme levels and ratios between the EULAR improvement group and the non-improvement group of RA patients using biologics.

	B-Cell ST3	B-Cell Neu3	B-Cell ST6	B-Cell Neu1	B-Cell ST3/Neu3	B-Cell ST6/Neu1
M0 − M3	-	-	-	-	-	-
M0 − M12	-	-	-	0.028	-	-
M0 − M15	-	-	-	-	-	-

*p*-values are shown; “-” indicates *p*-values > 0.05. ST3: α-2,3-sialyltransferase I; Neu3: neuraminidase 3; ST6: α-2,6-sialyltransferase I; Neu1: neuraminidase 1; ST3/Neu3: α-2,3-sialyltransferase I/neuraminidase 3 ratios; and ST6/Neu1: α-2,6-sialyltransferase I/neuraminidase 1 ratios. The EULAR improvement was calculated with the end-result DAS28-ESR < 3.2 and decreased scores of more than 1.2; the difference of enzyme levels between M0 minus M3 (M0 − M3), M0 minus M12 (M0 − M12), and M0 minus M15 (M0 − M15) was obtained and compared between the EULAR improvement and non-improvement group.

**Table 8:** Enzymes capable of discriminating RA activity improvement and fulfilling remission definitions.

Enzyme(s)	Fulfill the Criterion for Differentiating RA Activity Improvement	Fulfill Two Remission Definitions
B-cell Neu1	ACR, EULAR, and SDAI	Yes
B-cell ST6Gal1	ACR and SDAI	Only modified ARA remission
B-cell Neu3	SDAI	Yes
B-cell ST3Gal1	SDAI	Yes
Monocyte ST6Gal1/ Neu1 ratios	SDAI	Yes

RA: rheumatoid arthritis; Neu1: neuraminidase 1; ST6Gal1: α-2,6-sialyltransferase I; Neu 3: neuraminidase 3; ST3Gal1: α-2,3-sialyltransferase I; ACR: American College of Rheumatology; EULAR: European League Against Rheumatism; SDAI: Simplified Disease Activity Index; Modified ARA remission: 2005 modified American Rheumatism Association remission.

**Table 9:** Enzymes capable of discriminating RA activity improvement in subgroup categories.

Enzyme(s)	Fulfill the Criterion for Differentiating RA Activity Improvement in which Criterion/Subgroup
B-cell Neu1	ACR/RF, EULAR/RF, SDAI/RF; EULAR/anti-CCP, SDAI/anti-CCP; ACR/biologics, EULAR/biologics, SDAI/biologics
B-cell ST6Gal1	ACR/RF, SDAI/RF; ACR/anti-CCP, SDAI/anti-CCP
B-cell ST6Gal1/ Neu1 ratios	ACR/biologics
B-cell Neu3	EULAR/RF, SDAI/RF; SDAI/anti-CCP; SDAI/biologics
B-cell ST3Gal1	ACR/RF, EULAR/RF, SDAI/RF; SDAI/anti-CCP
Monocyte Neu1	SDAI/RF; SDAI/anti-CCP
Moncoyte ST6Gal1	SDAI/RF; ACR/anti-CCP, SDAI/anti-CCP
Moncyte ST6Gal1/ Neu1 ratios	SDAI/RF; SDAI/anti-CCP; ACR/biologics
Monocyte Neu3	ACR/RF, SDAI/RF; SDAI/anti-CCP
Monocyte ST3Gal1 /Neu3 ratios	ACR/anti-CCP; ACR/biologics, SDAI/biologics

RA: rheumatoid arthritis; Neu1: neuraminidase 1; ST6Gal1: α-2,6-sialyltransferase I; Neu 3: neuraminidase 3; ST3Gal1: α-2,3-sialyltransferase I; ST6: ST6Gal1. ACR: American College of Rheumatology; EULAR: European League Against Rheumatism; SDAI: Simplified Disease Activity Index; RF: rheumatoid factor; anti-CCP: anti-citrullinated peptide antibodies; biologics: use of biological agents.

## Data Availability

The data that support the findings of this study are displayed in the article and in the Supplementary Information. Other data are available from the corresponding author upon reasonable request.

## Supplementary Materials

The supporting information can be downloaded at: https://www.mdpi.com/article/10.3390/ijms241612998/s1.
