# Supplementary material for: Sialic-Acid-Related Enzymes of B Cells and Monocytes as Novel Markers to Discriminate Improvement Categories and to Fulfill Two Remission Definitions in Rheumatoid Arthritis
Source: Int J Mol Sci. 2023 Aug 20;24(16):12998. doi: 10.3390/ijms241612998 (PMC10455111; doi:10.3390/ijms241612998)
Supplement: Supplementary file 1 [file ijms-24-12998-s001.zip › ijms-2482227-supplementary.pdf]

[Supplementary Tables and Figures]

Sialic-acid-related enzymes of B cells and monocytes as novel markers to discriminate improvement categories and to fulfill two remission definitions in rheumatoid arthritis

Lieh-bang Liou, Ping-Han Tsai, Yao-Fan Fang, Yen-Fu Chen, Chih-chieh Chen, Jenn-Haung Lai

Supplementary Table S1. Comparing difference of B-cell and monocyte enzyme levels and ratios between the SDAI improvement group and the non-improvement group in RA patients with positive rheumatoid factor.

	B-cell ST3	B-cell Neu3	B-cell ST6	B-cell Neu1	Mono ST3	Mono Neu3	Mono ST6	Mono Neu1	B-cell ST3/Neu3	B-cell ST6/Neu1	Mono ST3/Neu3	Mono ST6/Neu1
M0–M3	0.038$^{\#}$	0.010$^{\#}$	-	-	-	-	-	-	-	-	-	-
M0–M12	<0.001*<br>0.007$^{\#}$	<0.001*<br>0.002$^{\#}$<br>0.045$^{@}$	<0.001*<br>0.045$^{\#}$<br>0.050$^{@}$	<0.001*<br>0.043$^{@}$	-	<0.001*	0.006*	<0.001*	-	-	-	0.001$^{@}$
M0–M15	-	-	-	-	-	0.036$^{\#}$	-	-	-	-	-	-

Shown are *P*-values; “-“ indicates *P*-values > 0.05. SDAI: Simplified Disease Activity Index; RA: rheumatoid arthritis; ST3: alpha-2,3-sialyltransferase I; Neu3: neuraminidase 3; ST6: alpha-2,6-sialyltransferase I; Neu1: neuraminidase 1; ST3/Neu3: alpha-2,3-sialyltransferase I/neuraminidase 3 ratios; ST6/Neu1: alpha-2,6-sialyltransferase I/neuraminidase 1 ratios; Mono: monocyte. The SDAI improvement was calculated with 50%*, 70%$^{\#}$ and 85%$^{@}$ reduction among groups with Month 0 minus Month 3 (M0–M3), M0 minus M12 (M0–M12), and M0 minus M15 (M0–M15). The non-improvement refers to those with less than 50%, 70%, and 85% reduction. Then, the difference of separate enzyme levels and ratios in fulfillment vs. non-fulfillment was compared to obtain *P*-values. All comparisons were done by Mann-Whitney U test.

Supplementary Table S2. Comparing difference of B-cell and monocyte enzyme levels and ratios between the SDAI improvement group and the non-improvement group in RA patients with positive anti-CCP antibodies.

	**B-cell ST3**	**B-cell Neu3**	**B-cell ST6**	**B-cell Neu1**	**Mono ST3**	**Mono Neu3**	**Mono ST6**	**Mono Neu1**	**B-cell ST3/Neu3**	**B-cell ST6/Neu1**	**Mono ST3/Neu3**	**Mono ST6/Neu1**
M0–M3	0.047$^{\#}$	0.032$^{\#}$	-	0.030$^{\#}$	-	-	-	-	-	-	-	-
M0–M12	0.005*	0.007*	0.003*	<0.001*	-	0.001*	0.040*	<0.001*	0.021*	-	-	0.008$^{@}$
	0.019$^{\#}$	0.005$^{\#}$		0.014$^{\#}$								
M0–M15	-	-	-	-	-	-	0.017$^{@}$	-	-	-	-	-

Shown are *P*-values; "-" indicates *P*-values > 0.05. Anti-CCP antibodies: anti-cyclic citrullinated peptide antibodies; all other abbreviations: see Supplementary Table S1. The SDAI improvement was calculated with 50%*, 70%$^{\#}$ and 85%$^{@}$ reduction among groups with Month 0 minus Month 3 (M0–M3), M0 minus M12 (M0–M12), and M0 minus M15 (M0–M15). The non-improvement refers to those with less than 50%, 70%, and 85% reduction. Then, the difference of separate enzyme levels and ratios in fulfillment vs. non-fulfillment was compared to obtain *P*-values. All comparisons were done by Mann-Whitney U test.

Supplementary Table S3. Comparing difference of B-cell and monocyte enzyme levels and ratios between the SDAI improvement group and the non-improvement group in RA patients with use of biologics.

	**B-cell ST3**	**B-cell Neu3**	**B-cell ST6**	**B-cell Neu1**	**Mono ST3**	**Mono Neu3**	**Mono ST6**	**Mono Neu1**	**B-cell ST3/Neu3**	**B-cell ST6/Neu1**	**Mono ST3/Neu3**	**Mono ST6/Neu1**
M0–M3	-	0.022[*]	-	0.037[*]	-	-	-	-	-	-	-	0.007[*]
M0–M12	-	-	-	0.022[#]	-	-	-	-	-	-	0.005[@]	-
M0–M15	-	-	-	-	-	-	-	-	-	-	-	-

Shown are *P*-values; “-“ indicates *P*-values > 0.05. All abbreviations: see Supplementary Table S1. The SDAI improvement was calculated with 50%*, 70%[#] and 85%[@] reduction among groups with Month 0 minus Month 3 (M0–M3), M0 minus M12 (M0–M12), and M0 minus M15 (M0–M15). The non-improvement refers to those with less than 50%, 70%, and 85% reduction. Then, the difference of separate enzyme levels and ratios in fulfillment vs. non-fulfillment was compared to obtain *P*-values. All comparisons were done by Mann-Whitney U test.

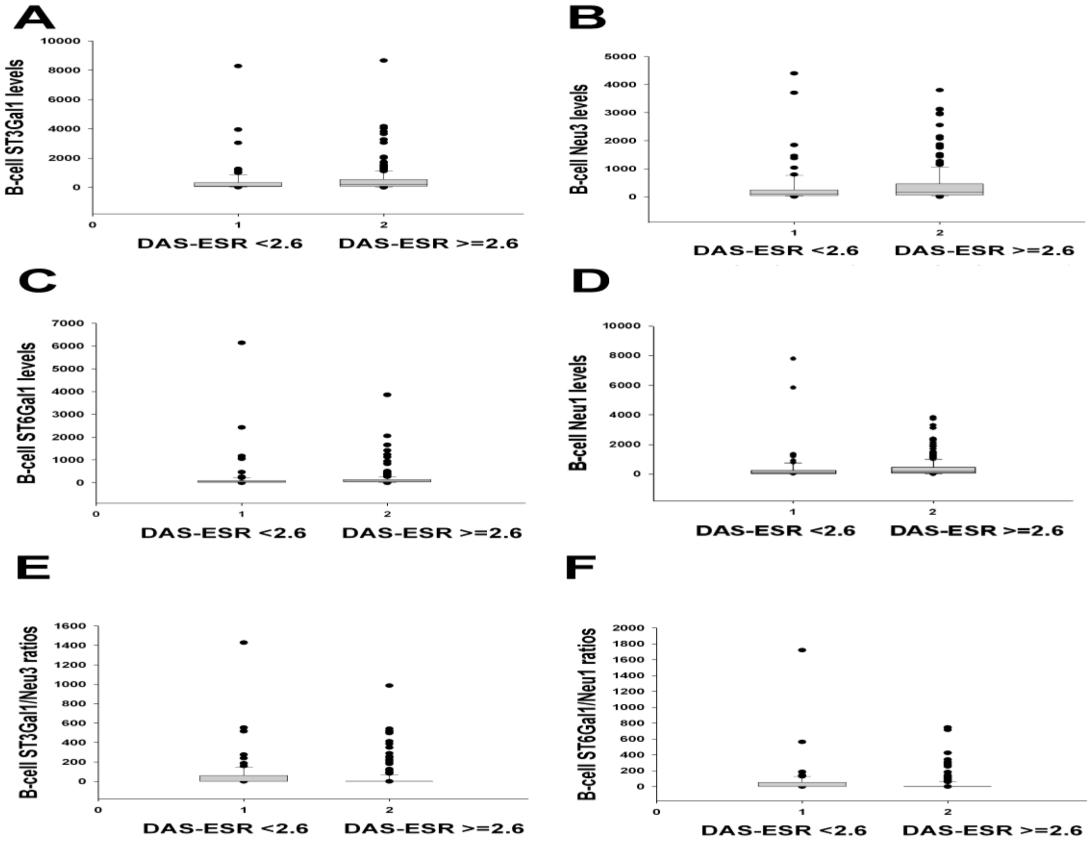

Supplementary Figure S1. Comparison of B-cell sialyltransferase and neuraminidase levels between DAS28-ESR remission and non-remission categories. All comparisons were analysed by Mann–Whitney U test. Visit numbers were 73 for DAS-ESR< 2.6 and 230-238 for DAS-ESR ≥ 2.6; the non-uniform latter is due to some smaller-group staining adjustment for the first several patients and some non-calculable ratios with zero enzyme levels as the denominator. (A) B-cell α-2,3-sialyltransferase I levels (B-cell ST3Gal1 level is the mean fluorescence intensity of cell enzyme staining) were compared between DAS-ESR< 2.6 and DAS-ESR ≥ 2.6 (higher): *P* = 0.005. (B) B-cell neuraminidase 3 (Neu3) levels were compared between DAS-ESR < 2.6 and DAS-ESR ≥ 2.6 (higher): *P* = 0.005. (C) B-cell α-2,6-sialyltransferase I levels (B-cell ST6Gal1 levels) were compared between DAS-ESR< 2.6 and DAS-ESR ≥ 2.6 (higher): *P* = 0.015. (D) B-cell neuraminidase 1 (Neu1) levels were compared between DAS-ESR< 2.6 and DAS-ESR ≥ 2.6 (higher): *P* = 0.002. (E) B-cell ST3Gal1/Neu3 ratios were compared between DAS-ESR< 2.6 (higher) and DAS-ESR ≥ 2.6: *P* < 0.001. (F) B-cell ST6Gal1/Neu1 ratios were compared between DAS-ESR< 2.6 (higher) and DAS-ESR ≥ 2.6: *P* = 0.002.

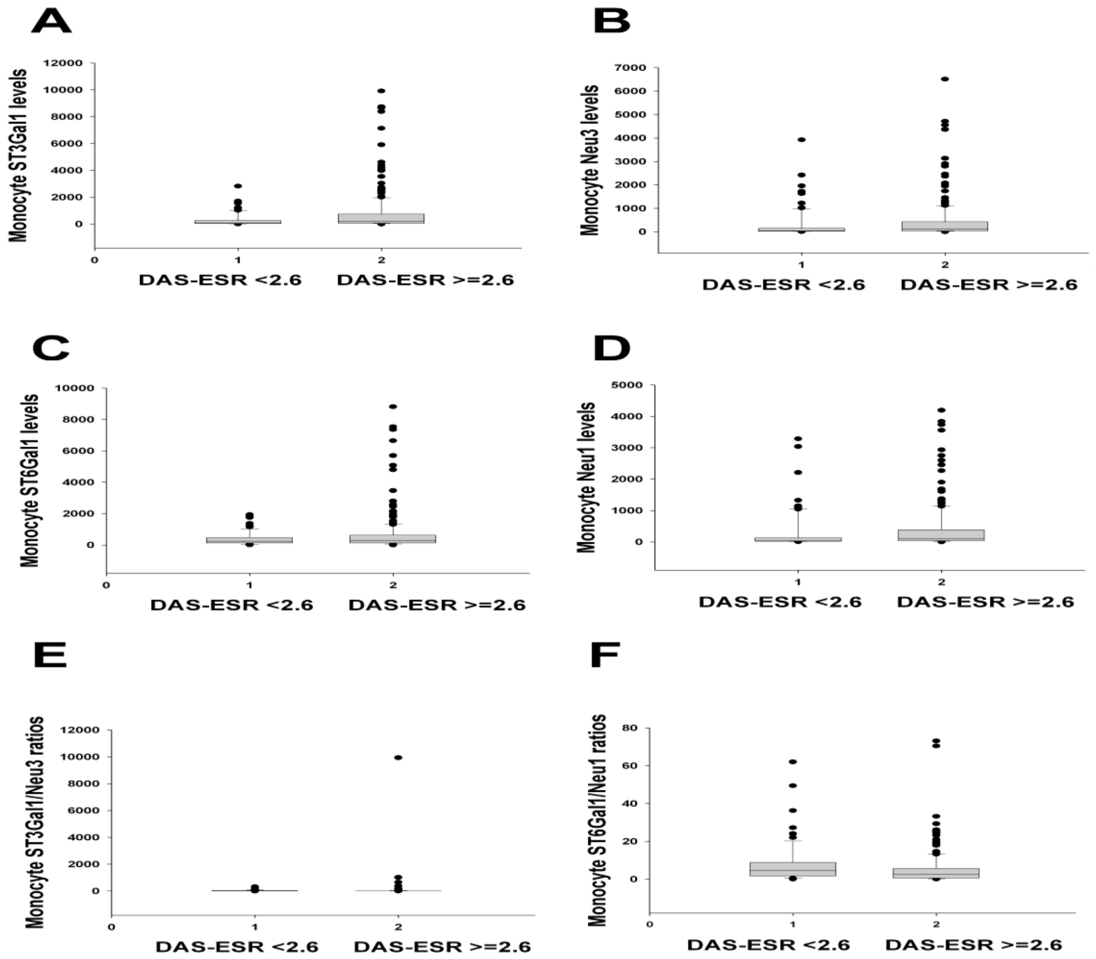

Supplementary Figure S2. Comparison of monocyte sialyltransferase and neuraminidase levels between DAS28-ESR remission and non-remission categories. All comparisons were analysed by Mann–Whitney U test. Visit numbers were 73 for DAS-ESR< 2.6 and 231-238 for DAS-ESR ≥ 2.6. (A) Monocyte α-2,3-sialyltransferase I levels (monocyte ST3Gal1 levels) were compared between DAS-ESR< 2.6 and DAS-ESR ≥ 2.6 (higher): $P < 0.001$. (B) Monocyte neuraminidase 3 (Neu3) levels were compared between DAS-ESR< 2.6 and DAS-ESR ≥ 2.6 (higher): $P = 0.001$. (C) Monocyte α-2,6-sialyltransferase I levels (monocyte ST6Gal1 levels) were compared between DAS-ESR< 2.6 and DAS-ESR ≥ 2.6 (higher): $P = 0.166$. (D) Monocyte neuraminidase 1 (Neu1) levels were compared between DAS-ESR< 2.6 and DAS-ESR ≥ 2.6 (higher): $P < 0.001$. (E) Monocyte ST3Gal1/Neu3 ratios were compared between DAS-ESR< 2.6 (higher) and DAS-ESR ≥ 2.6: $P = 0.004$. (F) Monocyte ST6Gal1/Neu1 ratios were compared between DAS-ESR< 2.6 (higher) and DAS-ESR ≥ 2.6: $P = 0.002$.

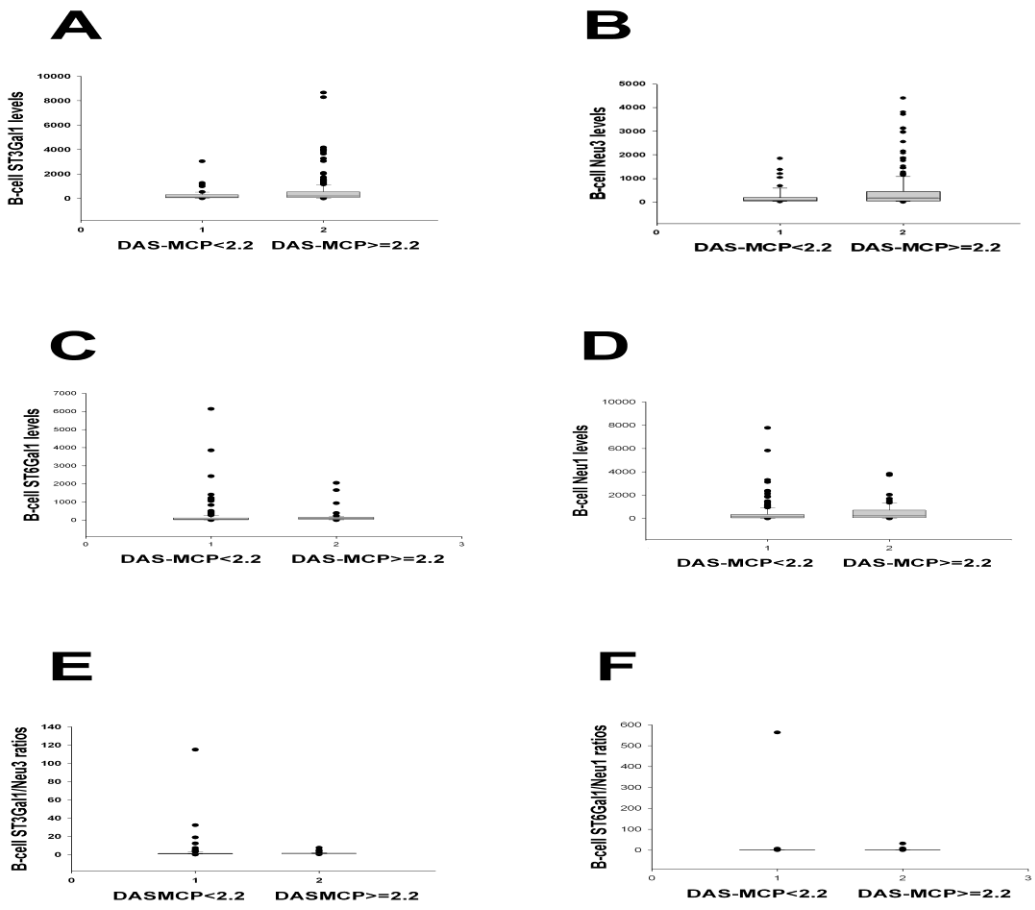

Supplementary Figure S3. Comparison of B-cell sialyltransferase and neuraminidase levels between DAS28-MCP-1 remission and non-remission categories. All comparisons were analysed by Mann–Whitney U test. Visit numbers were 55 for DAS-MCP-1 < 2.6 and 244-253 for DAS-MCP-1 ≥ 2.6; the non-uniform latter is due to some smaller-group staining adjustment for the first several patients and some non-calculable ratios with zero enzyme levels as the denominator. (A) B-cell ST3Gal1 levels (mean fluorescence intensity of cell enzyme staining) were compared between DAS-MCP < 2.2 and DAS-MCP ≥ 2.2 (higher): *P* = 0.009. (B) B-cell neuraminidase 3 (Neu3) levels were compared between DAS-MCP < 2.2 and DAS-MCP ≥ 2.2 (higher): *P* = 0.004. (C) B-cell ST6Gal1 levels were compared between DAS-MCP < 2.2 and DAS-MCP ≥ 2.2 (higher): *P* = 0.043. (D) B-cell neuraminidase 1 (Neu1) levels were compared between DAS-MCP < 2.2 and DAS-MCP ≥ 2.2 (higher): *P* = 0.001. (E) B-cell ST3Gal1/Neu3 ratios were compared between DAS-MCP < 2.2 and DAS-MCP ≥ 2.2: *P* = 0.174. (F) B-cell ST6Gal1/Neu1 ratios were compared between DAS-MCP < 2.2 and DAS-MCP ≥ 2.2: *P* = 0.845.

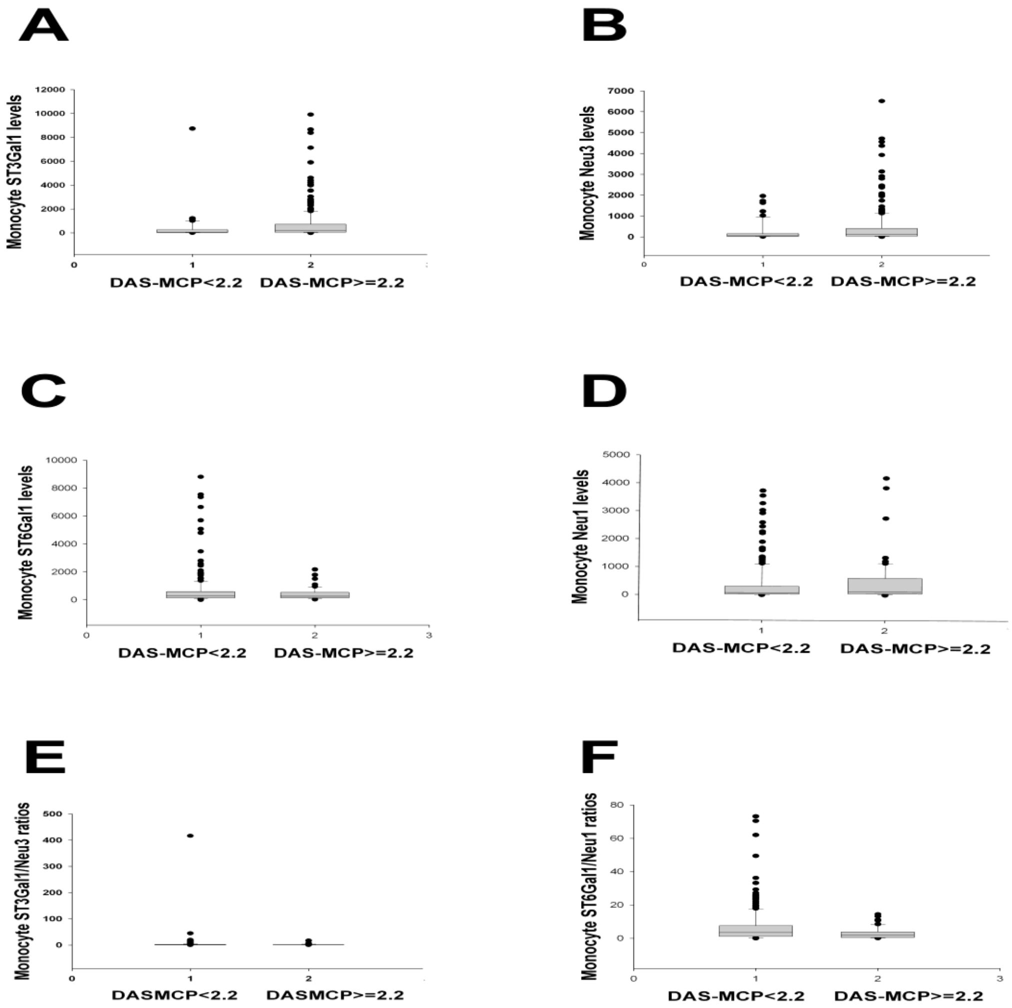

Supplementary Figure S4. Comparison of monocyte sialyltransferase and neuraminidase levels between DAS28-MCP-1 remission and non-remission categories. All comparisons were analysed by Mann–Whitney U test. Visit numbers were 55 for DAS-MCP-1 < 2.6 and 244-253 for DAS-MCP-1 ≥ 2.6. (A) Monocyte ST3Gal1 levels were compared between DAS-MCP < 2.2 and DAS-MCP ≥ 2.2 (higher): $P < 0.001$. (B) Monocyte neuraminidase 3 (Neu3) levels were compared between DAS-MCP < 2.2 and DAS-MCP ≥ 2.2 (higher): $P = 0.001$. (C) Monocyte ST6Gal1 levels were compared between DAS-MCP < 2.2 and DAS-MCP ≥ 2.2: $P = 0.718$. (D) Monocyte neuraminidase 1 (Neu1) levels were compared between DAS-MCP < 2.2 and DAS-MCP ≥ 2.2 (higher): $P = 0.001$. (E) Monocyte ST3Gal1/Neu3 ratios were compared between DAS-MCP < 2.2 and DAS-MCP ≥ 2.2: $P = 0.374$. (F) Monocyte ST6Gal1/Neu1 ratios were compared between DAS-MCP < 2.2 (higher) and DAS-MCP ≥ 2.2: $P = 0.003$.

Supplementary Table S4. Individual data for those hard-to-visualize differences clearly in Figures S1 to S2.

	**Number**	**25th percentile**	**Median**	**75th percentile**
Fig. S1C: B-cell ST6 for DASESR<2.6	73	18.65	40.40	86.05
Fig. S1C: B-cell ST6 for DASESR≧2.6	238	30.75	64.70	143.25
Fig. S1D: B-cell Neu1 for DASESR<2.6	73	39.45	89.40	236.50
Fig. S1D: B-cell Neu1 for DASESR≧2.6	238	62.75	184.00	448.50
Fig. S1E: B-cell ST3Gal1/Neu3 for DASESR<2.6	73	1.00	1.40	60.15
Fig. S1E: B-cell ST3Gal1/Neu3 for DASESR≧2.6	238	0.70	1.10	2.10
Fig. S1F: B-cell ST6Gal1/Neu1 for DASESR<2.6	73	0.30	0.60	53.35
Fig. S1F: B-cell ST3Gal1/Neu3 for DASESR≧2.6	238	0.20	0.40	1.60
Fig. S2E: Monocyte ST3Gal1/Neu3 for DASESR<2.6	73	1.15	2.20	31.50
Fig. S2E: Monocyte ST3Gal1/Neu3 for DASESR≧2.6	238	0.90	1.40	3.10

Data under percentile and median columns indicate mean staining fluorescence levels.

Supplementary Table S5. Individual data for those hard-to-visualize differences clearly in Figures S3 to S4.

	**Number**	**25th percentile**	**Median**	**75th percentile**
Fig. S3C: B-cell ST6Gal1 for DASMCP<2.2	250	21.38	55.55	121.25
Fig. S3C: B-cell ST6Gal1 for DASMCP≧2.2	59	40.20	76.60	160.00
Fig. S3E: B-cell ST3Gal1/Neu3 for DASMCP<2.2	243	0.760	1.070	1.460
Fig. S3E: B-cell ST3Gal1/Neu3 for DASMCP≧2.2	55	0.920	1.150	1.650
Fig. S3F: B-cell ST6Gal1/Neu1 for DASMCP<2.2	250	0.138	0.350	0.770
Fig. S3F: B-cell ST6Gal1/Neu1 for DASMCP≧2.2	57	0.145	0.340	0.940
Fig. S4C: Monocyte ST6Gal1 for DASMCP<2.2	250	126.75	296.00	579.25
Fig. S4C: Monocyte ST6Gal1 for DASMCP≧2.2	58	137.50	255.00	534.00
Fig. S4E: Monocyte ST3Gal1.Neu3 for DASMCP<2.2	243	0.960	1.370	2.480
Fig. S4E: Monocyte ST3Gal1.Neu3 for DASMCP≧2.2	55	0.860	1.250	2.220

Data under percentile and median columns indicate mean staining fluorescence levels.
